# Characterization and Differential Gene Expression between Two Phenotypic Phase Variants in *Salmonella enterica* Serovar Typhimurium

**DOI:** 10.1371/journal.pone.0043592

**Published:** 2012-08-24

**Authors:** Sheila K. Patterson, Klaudyna Borewicz, Timothy Johnson, Wayne Xu, Richard E. Isaacson

**Affiliations:** 1 Department of Pathobiology, University of Illinois, Urbana, Illinois, United States of America; 2 Department of Veterinary and Biomedical Sciences, University of Minnesota, St. Paul, Minnesota, United States of America; 3 Minnesota Supercomputing Institute, University of Minnesota, St. Paul, Minnesota, United States of America; East Carolina University School of Medicine, United States of America

## Abstract

*Salmonella enterica* serovar Typhimurium strain 798 has previously been shown to undergo phenotypic phase variation. One of the phenotypes expresses virulence traits such as adhesion, while the other phenotype does not. Phenotypic phase variation appears to correlate with the ability of this strain to cause persistent, asymptomatic infections of swine. A new method to detect cells in either phenotypic phase was developed using Evans Blue-Uranine agar plates. Using this new assay, rates of phenotypic phase variation were obtained. The rate of phase variation from non-adhesive to adhesive phenotype was approximately 10^−4^ per cell per generation while phase variation from the adhesive to the non-adhesive phenotype was approximately 10^−6^ per cell per generation. Two highly virulent *S.* Typhimurium strains, SL1344 and ATCC 14028, were also shown to undergo phase variation. However, while the rate from adhesive to non-adhesive phenotype was approximately the same as for strain 798, the non-adhesive to adhesive phenotype shift was 37-fold higher. Differential gene expression was measured using RNA-Seq. Eighty-three genes were more highly expressed by 798 cells in the adhesive phenotype compared to the non-adhesive cells. Most of the up-regulated genes were in virulence genes and in particular all genes in the *Salmonella* pathogenicity island 1 were up-regulated. When compared to the virulent strain SL1344, expression of the virulence genes was approximately equal to those up-regulated in the adhesive phenotype of strain 798. A comparison of invasive ability demonstrated that strain SL1344 was the most invasive followed by the adhesive phenotype of strain 798, then the non-adhesive phenotype of strain 798. The least invasive strain was ATCC 14028. The genome of strain 798 was sequenced and compared to SL1344. Both strains had very similar genome sequences and gene deletions could not readily explain differences in the rates of phase variation from non-adhesive to the adhesive phenotype.

## Introduction

*Salmonella enterica* is one of the most common causes of food borne diarrheal disease. The Centers for Disease Control has estimated that *S. enterica* is responsible for over a million cases of food borne illness per year in the United States [1]. *S. enterica* has been ranked as the leading cause of food borne disease as measured by the combined cost of illness and Quality Adjusted Life-Year [2]. The two most common serotypes associated with food borne illnesses are Typhimurium and Enteritidis. *S. enterica* serovar Typhimurium (*S.* Typhimurium) is commonly found in pigs and contaminated pork products have been a source of foodborne illnesses. One way that *S.* Typhimurium is believed to enter the food chain is by first establishing long-term persistent infections of pigs. Persistent infections are easily established early in life [3], [4] and once established *S.* Typhimurium can persist for the life of the animal. Persistently infected pigs intermitently shed *S.* Typhimurium in feces in low numbers (10–100 cells per gram of feces) [5]. It is believed that carrier animals are a major reservoir of *S. enterica* and may result in the contamination of foods during slaughter and processing [5], [6], [7], [8]. Even though pigs show no signs of infection or disease and may only shed the organism sporadically, stresses including transport and feed withdrawal promote the resumption of fecal shedding just prior to slaughter [5] and may be responsible for spread to uninfected animals held in lariage. Thus, swine can act as a reservoir for the spread of *S. enterica* throughout the herd, within the packing plant, and during processing to finished product.

Previously a clinical isolate of *S.* Typhimurium, that causes persistent infections in swine [3] was used to study the mechanism of persistence. This isolate, *S.* Typhimurium strain 798, was shown to exist in two distinct phenotypes designated adhesive and non-adhesive based on adhesiveness to porcine enterocytes [9]. Adhesive cells were shown to produce type 1 fimbriae that presumably were responsible for their adhesive properties, while non-adhesive cells did not [10]. Cells in one phenotype could convert to the other phenotype at a rate of approximately 10^−4^ per cell per generation. This high rate of transition between phenotypes led us to believe that the transition was due to phase variation and not mutations. For simplicity, we refer to adhesive phase cells as i519 and non-adhesive phase cells as i518. These designations are not meant to indicate that they are separate strains. Analysis of crude envelope protein preparations by SDS-PAGE and Western blot showed that i519 cells displayed at least 10 unique proteins and 4 unique antigens when compared to i518 cells [9]. i519 cells also were more easily phagocytosed by porcine leukocytes and were able to survive and replicate in porcine leukocytes after phagocytosis, while i518 cells were less readily phagocytosed but once in phagocytes were rapidly killed [9]. i519 cells have a long O-antigen (1–17 repeats) while i518 cells have a short O-antigen (2–4 repeats), and the long O-antigen also confers resistance to complement [11]. Because all of these traits were coordinately expressed, we assume that a single regulatory mechanism controls these traits. Strain 798 is unique compared to highly virulent strains because it has a high oral LD_50_ (∼10^9^) in BalbC mice [10]. This is 4–5 orders of magnitude higher than typical virulent *S.* Typhimurium strains such as SL1344 or ATCC 14028. When pigs were infected orally with 10^9^ *S.* Typhimurium strain 798 cells, about half of the pigs challenged had mild, transient diarrhea while the other half experienced no disease [5]. All pigs became asympotomatic carriers suggesting that low virulence strains cause persistent, sub-clinical infections.

The pathogenesis of *S.* Typhimurium has been intensively studied and many virulence factors have been identified [12], [13], [14], [15], [16], [17], [18], [19], [20], [21], [22]. However, few studies have addressed the question of how persistent infections are established and maintained in animals. We believe that persistence is the result of prolonged colonization of mucosal surfaces or through oral-fecal reinoculation or both. We hypothesize that phase variable expression of specific virulence genes, including adhesins and invasins, contribute to the development and maintenance of persistent infections and are important in keeping cell numbers low thus preventing clinical disease yet maintaining a stable population.

The work described here was performed to determine if phenotypic phase variation was unique to strain 798 or if high virulence strains of *S.* Typhimurium also under go phenotypic phase variation. A simple method to measure phase variation using Evans Blue Uranine agar (EBU) plates was developed for this purpose. In addition, the extent of differential gene expression between the two phenotypes was determined and compared with the virulent *S.* Typhimuiurm strain SL1344 using using RNA-Seq. Finally, the genome sequence of strain 798 also was determined and compared to the sequence of strain SL1344 to identify genomic sequences that might contribute to phenotypic differences between strains 798 and SL1344.

## Results

### Evans-blue Uranine Plates Differentiate Phase Variants

**Figure 1:**
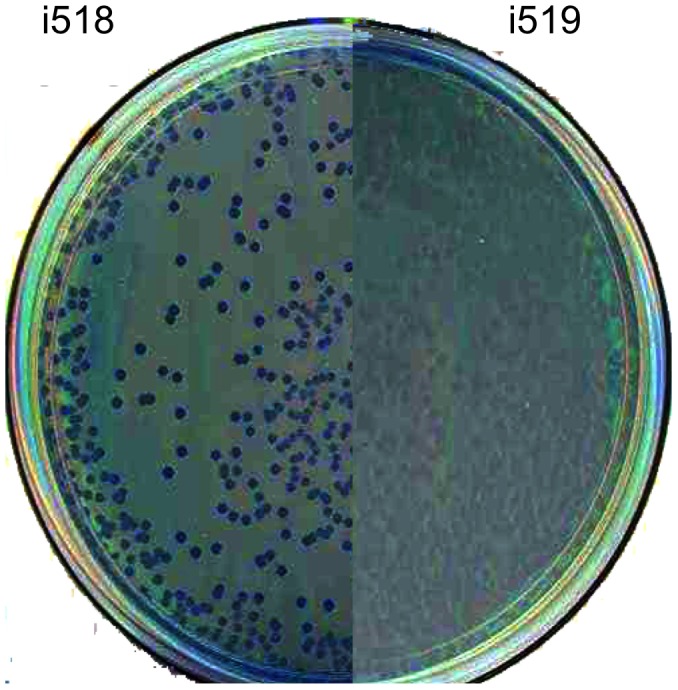
Image of i518 and i519 colonies on an Evans Blue Uranine agar plate. Colonies of i518 are dark and colonies of i519 are light.

Cells in the adhesive phenotype, i519, have been shown to produce type 1 fimbriae, while cells in the non-adhesive phenotype, i518, do not [9], [23]. In our previous studies to compare the two phenotypes, we created a knockout of type 1 fimbrial production by moving a *fimA*::Tn*phoA* fusion from i519 (mutant #14 [23]) into i518 using P22 transduction. After bacteriophage transduction it is customary to purify colonies on Evans blue-Uranine (EBU) plates [24] to eliminate pseudolysogens. Colonies containing bacteriophage infected cells appear dark blue while colonies free of bacteriophages are light-colored. Following transduction of *fimA*::Tn*phoA* using bacteriophage P22 with i518 as the recipient, cells were streaked to EBU. It was found that i518- *fimA*::Tn*phoA* colonies on EBU were mainly dark blue in color suggesting the presence of P22 in these cells. Only occasionally did i518 colonies produce light-colored colonies. This result was in contrast to P22 transduction into i519, which were found to produce mainly light-colored colonies. Because the relative population of i518 transductants that remained blue was quite high, we wondered whether this was due to a phenotypic difference between i518 and i519 cells rather than indicating the presence of bacteriophages in i518. Thus, i518 cells from a freezer stock that had never been infected with P22 were streaked to EBU. The colonies from the frozen culture stocks of i518 produced mainly blue colored colonies with a few light colored colonies. An image showing the color differences between the two phenotypes, i518 and i519, when grown on EBU plates is shown in Figure 1.

A yeast cell agglutination assay was used to determine if the light colonies from i518 were actually phase variants and produced type 1 fimbriae compared to the blue colored colonies. Yeast cells did not agglutinate cells from blue-colored i518 colonies, whereas cells from light-colored i519 colonies did agglutinate and this agglutination was inhibited by D-mannose. Light-colored colonies derived by plating i518 on EBU agar gained the ability to agglutinate the yeast solution. This agglutination was inhibited by D-mannose. Blue colonies also were found after plating a single light-colored i519 colony on EBU plates. These colonies did not agglutinate yeast cells. These data indicate that the light colored cells produced type 1 fimbriae, while the dark colored colonies did not and that color on EBU plates will be useful in tracking phase variation.

### Measuring Rates of Phase Variation

Because colony color differences on EBU plates can distinguish cells in the two phenotypes, we used the color of colonies to calculate rates of phase variation between i518 and i519. The weighted rate takes into consideration the number of colonies screened. To calculate the rates, the assumption was made that light-colored colonies arose from a single light-colored parent and blue colonies arose from a single blue parent [25]. Blue colonies that had a light section comprising greater than 25% of the colony were counted as phase variants. From this data the calculated weighted rate of phase variation from i518 to i519 was 1.7×10^−4^ per cell per generation (Table 1). The rate of phase variation for i519 to i518 could not be calculated in this manner because the rate was approaching the detection limit of the screen at less than 10^−5^ per cell per generation.

**Table 1:** Rate of phenotypic phase variation between different strains of *S.* Typhimurium.

Strain	On to Off	Off to On
798	1.5×10^−6^	1.7×10^−4^
SL1344	3.0×10^−6^	3.2×10^−2^ *
14028	4.8×10^−6^	1.2×10^−2^ *
LT2	Not detectable	Not determined

* Significant at ≤0.03 compared to strain 798.

**Figure 2:**
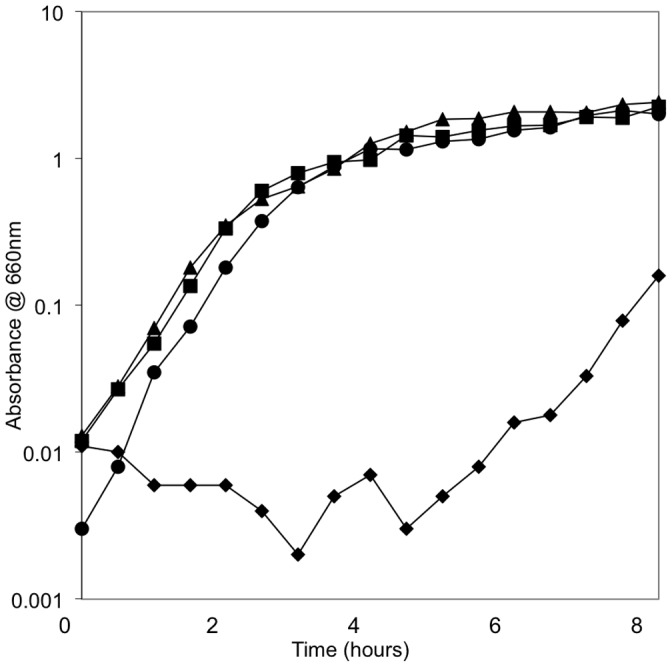
Growth of i518 and i519 in LB With 1% Salt and LB Without Added Salt. Growth curves of i518 and i519 were repeated on three separate occasions. Spectrophotometric readings were taken every thirty minutes. i518 grown in LB-0: ⧫ i518 grown in LB-H: ▴, i519 grown in LB-0: ▪, i519 grown in LB-H: • Shown is a single representative graph.

While attempting to determine if there were other differences between the two phenotypes being studied, we performed growth experiments using cells in both phenotypes and comparing the effects of NaCl on growth. This experiment was based on the findings of others who found that osmolarity regulated certain virulence genes in *S. enterica* [26], [27]. No differences in growth rate in exponential phase, length of lag phase, or cell concentration where stationary phase occurred were observed when growth was in LB-H broth (1% NaCl). In the next set of growth experiments we used LB-0 that did not contain NaCl. Growth of i518 in LB-0 resulted in a much longer lag phase before reaching exponential phase of growth. Growth of i519 in LB-0 grew the similarly to i519 growth in LB-H (Figure 2). The length of the lag phase for i518 varied and ranged between 2.5 hours and 7.5 hours. To determine if prolongation of lag phase was due to i518 cells being unable to grow, viable cell counts were determined at 60 minute intervals during the growth experiment by plating on EBU agar. Following incubation at 37°C, colonies were counted and the percentages that were blue were calculated. In a typical experiment, it was found that the viable cell count of i518 dropped in the first half of the lag period and then began to increase steadily. The percentage of cells that had a blue colony color on EBU dropped steadily from almost 100% to almost 0% by the time exponential phase of growth was reached. i518 cells grown in LB-H did not experience a drop in viable cell count nor did the percentage of blue colonies on EBU change. This data indicates that when i518 cells were grown in LB-0 the i519 phenotype was selected. These results also suggest that the shift of i518 cells to the i519 phenotype could be manifested through decreased viability of the i518 phenotype under low ionic conditions.

The differential sensitivity observed in LB-0 was exploited to develop a procedure to determine the relative rate of phase variation of i519 phenotype to i518. An ampicillin enrichment procedure was used for this purpose. Ampicillin enriched for cells in the non-adhesive phenotype since i518 blue colonies did not actively grow in LB-0. We reasoned that ampicillin would kill the actively growing i519 phase cells but would not affect the non-growing i518 phase cells. If the exposure to ampicillin was brief enough, the i518 cells in LB-0 would remain viable. A single colony of i519 was incubated in 3 ml of LB-0 that contained ampicillin (100 ug/ml) for two hours at 37°C. Diluting and plating this mixture on EBU plates allowed us to enrich for phase variants. An incubation time of two hours was chosen in a pilot study because i518 (blue colonies) cells remained at approximately the same viable count after incubation in LB-0 for 2 hours. However, incubation in the presence of ampicillin resulted in a ten-fold reduction in the number of viable i519 cells. Thus, we were not incubating so long as to lose any potential phase variants but were gaining a ten-fold enrichment which allowed us to calculate a relative rate of phase variation. The relative weighted rate was then calculated and found to be 1.5×10^−5^ per cell per generation. However, when the 10-fold increase in sensitivity was added to the equation, the rate was estimated to be 1.5×10^−6^ per cell per generation (Table 2).

**Table 2:** *Salmonella enterica* serovar Typhimurium strains used.

Strain	Phenotype/genotype	Source
798	Wild type from a pig	[3]
i518	Non-adhesive phenotype of 798	[9]
i519	Adhesive phenotype of 798	[9]
Mutant 14	Strain 798 with Tn*PhoA*::*fimA*	[23]
SL1344	High virulence strain	[47] From J. Slauch
14028	High virulence strain	ATTC
LT2	Laboratory strain	ATTC
14028-hilA	*HilA* knockout	[26] From J. Slauch

Finally, we wanted to know whether strain 798 was unique exhibiting the described phenotypic phase variation. Therefore, the rate of phase variation of two highly virulent *S.* Typhimurium strains SL1344 and ATCC 14028 were determined using the same procedures. The estimated rates of phase variation to the non-adhesive phenotype (blue colonies on EBU) was similar to what we measured using i519 (Table 2). However, cells in the non-adhesive phenotype (blue colonies on EBU) transitioned back to the adhesive phenotype (light colonies on EBU) at significantly higher rates (p = 0.03) compared to i518: approximately 37-fold higher. Thus, we concluded that while the virulent *S.* Typhimurium strains SL1344 and ATCC 14028 also could phase vary, they were more likely to be in the adhesive phase (light colony) phenotype because of the high rate of of phase variation from blue to light colonies. *S.* Typhimurium strain LT2 also was used in these experiments and phase variation could not be detected. All colonies of LT2 remained light on EBU plates. We concluded that strain LT2 does not phase vary in the way that the other strains do.

### Differential Gene Expression

Because the two phenotypes of strain 798 have important virulence based differences including invasiveness, adhesiveness, intracellular survival in macrophages and neutrophils, O-antigen chain length, and serum sensitivity and because these phenotypic characteristics were coordinately co-regulated, we hypothesized that other genes could be part of this phenotypic repertoire. Consequently, global gene expression of i518 and i519 cells was determined and compared using RNAseq. In addition, *S.* Typhimurium strain SL1344 was used in this comparison as an example of a high virulence strain. SL1344 cells were in the adhesive/light color phenotype. The results of these comparisons are shown in Tables S1 and S2. Differential expression was defined as having in excess of 2-fold increase or decrease in gene specific mRNA levels after normalization and a statistical probability value of <0.05. Using these criteria 83 genes were identified as being up-regulated in i519 compared to i518 Table S1) and 31 genes (were identified as being down regulated. To validate the RNA-Seq results, RT-PCR was performed using 13 up-regulated genes, 3 down-regulated genes and 5 genes that were not differentially regulated. Using the Spearman rank correlation test to compare the RT-PCR results with the RNA-Seq results it was found that the two procedures were statistically comparable.

**Figure 3:**
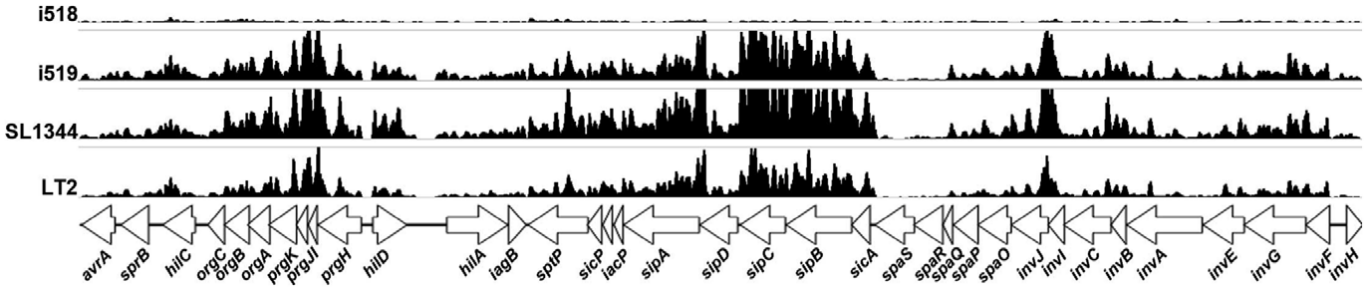
Comparison of expression of the SPI1 genes. RNA-Seq data representing results from *S. enterica* i518, 1519, SL1344, and LT2 are graphically compared and aligned with the known SPI1 genes.

Included in the set of genes that were up-regulated were all genes in the Salmonella pathogenicity island 1 (SPI1), effector molecules such as *sopA*, *sopB*, *sopD*, and *sopE*, several type three secretion related genes of Salmonella pathogenicity island 2, genes involved in chemotaxis, and genes involved in propandiol utilization (*pduA, B, C, D, E, and H*). Figure 3 shows the expression profile comparison for the SPI1 genes. Within the group of down regulated genes were superoxide dismutase and catalase. Two other genes, *osmC* and *osmY* that encode proteins related to a membrane inducible salt-shock response, also were down regulated. The salt-shock inducible genes could be related to the observed phenotypic sensitivity of i518 cells in LB-0.

A comparison of i519 cells to SL1344 cells identified 37 genes that were up-regulated in i519 and 35 genes that were down-regulated (Table S2). None of these genes were those identified in the comparison of i518 and i519. Of the up-regulated genes, 4 could be associated with virulence: repressor of phase 1 flagellin, bacterioferritin, phase 2 flagellin, and resistance to quaternary ammonium compounds. The remainder of the up-regulated genes were classified as metabolic pathways or regulatory genes. The genes that were down regulated in i519 compared to SL1344 were mainly classified as metabolic with ethanolamine utilization a major down-regulated pathway. Also of note was the down-regulation of the minor subunit of curli, which might be important in biofilm formation [28]. As another comparison of data, expression of SL1344 was compared to i518 cells (Table S3).

### Invasion Assays

**Figure 4:**
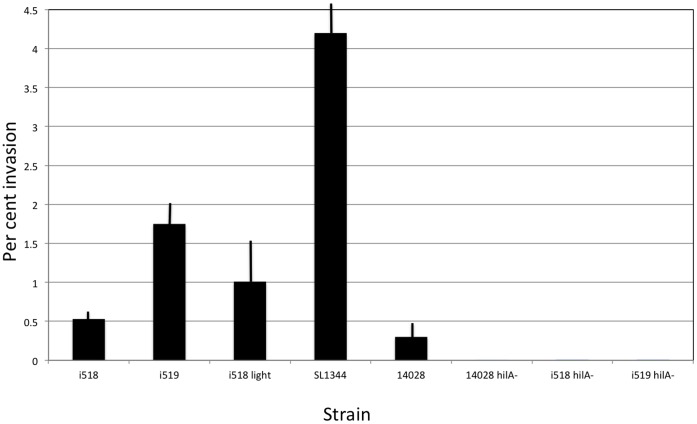
Results of invasion assays using Henle 407 cells. Invasion assays were performed using the strains indicated. All final results represent the mean of 3 replicates. Error bars are +/− one standard deviation. The data is expressed as the percent of bacterial cells that invaded the Henle 407 cells. The *hilA* mutants were each used as negative controls as HilA is required for expression of genes involved in invasion.

Invasion assays were performed using a gentamicin protection assay [29] and Henle 407 cells. The results are shown in Figure 4. i518 cells invaded poorly compared to i519 (only 27%). This result showed statistical significance (p = 0.0008). This result is similar to what we previously saw using porcine leukocytes [9]. A light-colored adhesive phase variant of i518 was picked after plating on EBU plates and was shown to regain the ability to invade the Henle 407 cells (77% when compared to i519: p = 0.14). The i518 light-colored phase variant invaded significantly better than the blue i518 phenotype (p = 0.02). Next, two highly virulent *S. *Typhimurium strains were tested. *S.* Typhimurium strain ATCC 14028, was less invasive than i518 and i519 phenotypes. On the other hand, strain SL1344 was more invasive than i519 cells. As negative controls, *hilA* mutants of ATCC 14028 (courtesy of J. Slauch), i518 and i519 were tested for invasiveness. All three were non-invasive.

### Genome Sequencing of *S.* Typhimurium Strain 798

**Figure 5:**
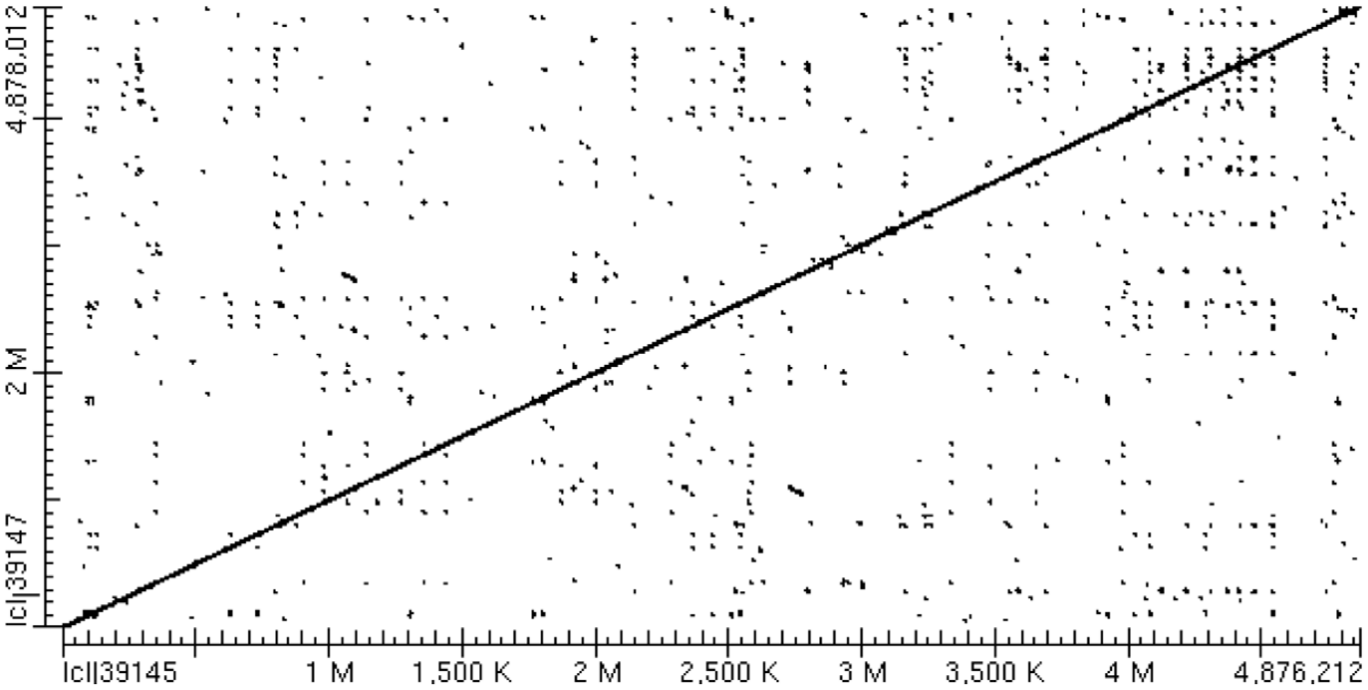
Alignment and comparison of the genomes of *S.* Typhimurium strains 798 and SL1344. The results are visualized using a dot plot prepared using BLAST.

In an attempt to further understand the mechanism mediating phenotypic variation of strain 798 we sequenced the genome of this strain. Sequencing was performed using a Roche 454 GS sequencer using FLEX chemistry and the sequence was assembled using Newbler assembler. To assist in the assembly process, we used the finished sequence of strain SL1344 as a scaffold. The use of the Newbler assembler resulted in the formation of 80 contigs of the chromosome and 2 contigs of the large plasmid. The sequence was closed using PCR amplification and conventional Sanger sequencing. The assembled chromosomal sequence was then aligned with the published sequence of SL1344 using BLAST. Strain 798 was shown to be high similarity to SL1344 as demonstrated using Mauve and BLAST. An alignment dot plot is shown in Figure 5. The chromosome of strain 798 was shown to contain 4,876,219 base pairs which is 1,793 base pairs smaller than the chromosome of SL1344. Only four differences involving insertions/deletions were identified between 798 and SL1344, including insertions in STM1009, *ygbM*, and an intergenic region spanning *iap* and *ygbF* in 798; and an insertion in *bigA* in SL1344. Because genes in SPI1 are all up-regulated in i519 compared to i518, we asked if the SPI1 sequence in strain 798 differed from that in SL1344. We identified 160 single nucleotide polymorphisms within the core sequences of the entire genomes of 798 and SL1344, but none of these were within SPI1. Of the genomic SNPs identified, 30 involved intergenic regions and the remainder were found within coding regions. A number of SNPs were identified throughout the genome that were located in virulence genes or transcriptional regulators.

## Discussion

This study was undertaken to investigate the differences between the two phenotypic variants of *S.* Typhimurium strain 798. In the process we found an easy method to differentiate the two phases. The method involved the use of EBU plates, which are typically used to detect pseudolysogens after transduction. The two phase variants exhibited very different colony morphologies and color when plated on EBU. The color differences on EBU plates were coordinate with the expression of type 1 fimbriae as determined by yeast cell agglutination assay.

Using the differential color response on EBU plates, the rates of phase variation were measured between the two phenotypes. Previously, it was estimated that the rate of phase variation from the non-adhesive to the adhesive phenotype was approximately 10^−2^ per cell per generation and the reverse direction was approximately 10^−4^ per cell per generation [9]. However, those rates were based on observed differences in colony morphology when plated on Tryptone phosphate broth. The current study provides estimates of the rates of phase variation based on the use of EBU plates and showed that non-adhesive phase cells actually phase vary to adhesive phase cells at a rate of approximately 1.7×10^−4^ per cell per generation. The adhesive phase cells phase varied to the non-adhesive phase at a relative weighted rate of 1×10^−6^ per cell per generation. Why the measured rates of phase variation differed from our previous results is likely because effects associated with the use of different media. The differences in the forward and reverse rates of phase variation is not unusual as many systems, including the phase variation of Lpf and *E. coli* type 1 pili, occur at different rates between “off” to “on” and “on” to “off” [23], [25], [30], [31]. It is important to put these results in perspective as the rates of phase variation measured here are for unique aerobic in vitro conditions using specific culture media. How these rates may relate to in vivo conditions are not known.

The highly virulent strains SL1344 and ATCC 14028 also were shown to undergo phase variation to blue-colored colonies on EBU (the non-adhesive phenotype) demonstrating that the described phase variation process was not restricted to strain 798. Using EBU plates, the rates of phase variation to blue colony type of both were similar to that of i519 adhesive phase cells varying to the i518 non-adhesive phase. However, the rates of phase variation from blue to light-colored were much higher for SL1344 and ATTC 14028. There were two reasons why we selected strains SL1344 and ATCC 14028 for study. Firstly, these strains are frequently used in virulence-based studies and there is a significant database on their biological responses compared to strain 798. Secondly, strain 798 is a low virulence strain but can cause non-symptomatic persistent infections in pigs, which we have hypothesized is caused by a modulation of virulence gene expression through phase variation. It is not clear if SL1344 or ATCC 14028 could cause persistent infections of pigs. In our hypothesis of persistence, it is assumed that the virulent strains were not capable of persistent infections because they are maintained in a phenotype of high virulence and thus cause disease until the host is able to clear them. While cells that cause persistent, asympotomatic infections like strain 798 do so by remaining at a concentration below a threshold that induces a host response. Since a large fraction of the bacteria remain in the non-adhesive phenotype they are not able to colonize or invade host tissues and therfore are rapidly shed from the animal. The remaining cells that are in the adhesive or virulent phase represent a dose too low to exert clinical disease. Since adhesive phase cells always produce some non-adhesive phase cells, low cell numbers can be maintained. For the high virulence strains, the majority of cells remain in the virulent phenotype and therefore disease rather than persistence should result. If the rate of phase variation from the non-adhesive phase to the adhesive were lower perhaps these strains would be able to cause persistent, non-clinical infections. Since the conditions used to measure phase variation in all of the *S. enterica* strains tested were in vitro, our predictions of how each will be behave in vivo will require additional experiments.

Because the highly virulent strains SL1344 and ATCC 14028 also were capable of phase variation and because the rate of phase variation from non-adhesive to the adhesive phase was much higher than what was observed for strain 798, we wanted to know whether there also were differences in invasiveness of these strains. The data demonstrated that strain SL1344 was the most highly invasive strain in the in vitro assay followed by i519 cells, i518 cells, and then ATCC 14028 cells. It is known that SL1344 is more highly invasive when compared to strain ATCC 14028 [32] and it has been proposed that an effector protein that is present in SL1344 cells, SopE, is responsible for this difference. Cells of strain ATCC 14028 produce a homolog of SopE called SopE2. Our genomic analysis of strain 798 demonstrated that it also produced SopE and not SopE2. This might explain why i519 cells were more invasive. However, regardless of invasiveness and expression of SopE, compared to the two highly virulent strains, strain 798 was of low virulence (oral LD_50_ ∼ 10^9^).

Our previous data demonstrated that phase variation in *S. *Typhimurium strain 798 coordinately controlled expresssion of type1 fimbriae, invasiveness, intracellular survival, O-antigen chain length, and sensitivity to complement. Using RNA-Seq, we now show the entire repertoire of genes differentially expressed between cells in the two phenotypes grown in LB. The most highly up-regulated genes included all genes encoded in the *Salmonella* pathogenicity island 1. The most highly expressed gene was *sipC* and it was over expressed 104-fold in i519 compared to i518 while *spaS* was over expressed 2.7-fold in i519 compared to i518. Genes encoded by four effector molecules (SopA, SopB, SopD and SopE) secreted by the SPI1 type three secretion system were up-regulated (5–51-fold). The SPI1 genes are important for invasion of target cells and are part of the gene repertoire required for early establishment of infections. Also up-regulated were genes that encode propandiol utilization (3.3–2.2 fold). This system may be a metabolic adaptation to the in vivo life style of *S. enterica* during disease. We expected that genes involved in type 1 fimbrial expression also would be up-regulated. However, using the growth conditions selected for RNA-Seq, *fimA*, the gene encoding the major subunit of type 1 fimbriae, was not in the group of up-regulated genes. A closer look at the data showed that *fimA* expression was up-regulated approximately 2-fold, but did not satisfy the statistical criteron of a p value of <0.05. The reason why type 1 fimbrial expression was not up-regulated to a greater extent is not known. Perhaps additional replicates would have generated additional data that would then result in statistical significance. Because cell populations of both phenotypes are a mix of both phenotypes, its also possible that for these experiments the ratio of phenotypes was sufficient to bias the results. For example, if the cell mix in the non-adhesive phenotype were shifted slightly to having more adhesive cells, it could raise relative expression just enough to result in a non-statisticallly significant result. This could occur if expression of *fimA* relative to some of the other highly up-regulated genes like *sipC* is not as highly expressed. It is known for some *E. coli* fimbriae that small changes in fimbrial gene expression can result in large differences in fimbrial production [33]. We speculate that this outcome also could be the result of the in vitro growth conditions selected. A similar situation was observed for expression of catalase and *rpoS.* Catalase was in the down regulated catagory. Expression of *rpoS,* which has been correlated with induction of catalase expression [34], was not down-regulated in strain 798.

The most obvious differences in expression between i519 cells and SL1344 is the over expression of genes involved in ethanolamine utilization by SL1344 (5–15 fold). Ethanolamine utilization has recently been shown to be involved in virulence [35] and this difference between the two strains may explain in part why SL1344 is of higher virulence compared to i519 cells.

Phenotypic phase variation has been known to occur in many pathogens and several different mechanisms have been identified that mediate the process. For example, phase variation of type 1 pili of *Escherichia coli* has been shown to be mediated by the inversion of a DNA fragment that contains the promoter responsible for expression of the pili [25]. Phase variation of *Neisseria gonorhoeae* pili is mediated by insertion of a unique pilin gene cassette into an expression site [36]. Differential methylation of GATC boxes by Dam methylase is a third mechanism of phase variation and controls expression of pyelonephritis associated pili [37]. Slipped-strand mispairing also has been identified as a fourth mechanism promoting phase variation [38]. In each of these cases, the effects of phase variation is limited to a small set of genes that appear to be involved in one aspect of pathogenesis: namely adhesion to mucosal surfaces. The phenotypic phase variation described here appears to control a much larger array of genes and processes. Currently, we have not identified the specific mechanism mediating this phase variation process. However, it is our hypothesis that phase variation is important in the establishment of chronic, but asymptomatic infections of animals by *S.* Typhimurium. Our hypothesis is that phase variation controls the relative fraction of cells in a virulent and avirulent phenotype and by establishment of the proper ratio between the two, permits low level colonization and invasion of the intestinal epithelium of animals, yet sets the level of cells that can invade below a threshold that would result in clinical disease. This would explain why strain 798 can maintain long term persistent colonization in the absence of disease while strains SL1344 and ATCC 14028 remain highly virulent and each have an oral LD_50_ that is relatively low. Our analysis of the genome of strain 798 was performed in part to determine if there were fundamental differences in this strain when compared to SL1344. A comparison of the sequences of these two strains has shown remarkable similarity. The chromosome of strain 798 is 1,793 base pairs shorter than in SL1344 and while this difference could represent the major difference between strains, we did not identify a unique gene(s) that was missing in 798. Likewise, the large virulence plasmid of 798 was nearly identical to the plasmid in SL1344. While there are numerous small differences between both genomes, we believe that reasons for differential expression of phase variation is more likely to be linked to small nucleotide alterations in non-translated DNA.

Overall, the work presented here is consistent with the hypothesis that phase variable expression of virulence genes is important in the development and maintenance of persistent infections and is important in keeping cell numbers low thus preventing clinical disease yet maintaining a stable population. The results from measuring the rates of phase variation of two high highly virulent strains and strain 798 showed that the two highly virulent strains were more likely to be found in the highly virulent phenotype compared to strain 798. Thus, since strains SL1344 and ATCC 14028 are more likely to be of the virulent phenotype they are more likely to cause acute disease while strain 798 is more likely to be a mixture of the two phenotypes and thus cause low grade or subclinical infections. i519 cells were shown to be more invasive than i518 cells and both were more invasive than the virulent strain ATCC 14028. Strain SL1344, however, was the most invasive of the strains tested. In addition, we used RNA-Seq to measure differential gene expression and found that i519 had much higher expression of numerous virulence related genes when compared to i518 cells. Expression of these virulence genes did not differ from highly virulent cells of strain SL1344.

## Materials and Methods

### Bacterial Strains and Growth Conditions

Bacterial strains used in this study are listed in Table 2. Bacterial cultures were shaken at 37°C in Tryptone Phosphate Broth (TPB) (Gibco-BRL) except where noted. Low osmolarity media (LB-0) consisted of Luria-Bertani broth (LB) with no added NaCl (0.5% Bacto-yeast extract, 1% Bacto-tryptone). High osmolarity media (LB-H) consisted of LB with 1% NaCl. Static cultures were grown without shaking at 37°C. Evans Blue-Uranine (EBU) plates were prepared as previously described [24]. Bacterial cultures were routinely plated on EBU plates to determine which phenotypic phase was present.

### Yeast Cell Agglutination Assay

*Saccharomyces cerevisae* cells can be used to detect type 1 fimbriae by virtue of mannose being present on their cell surfaces. *S. cerevisae* cells were obtained in the form of Bakers’s Yeast and suspended in PBS to a final concentration of 3%. A drop of the yeast cell suspension was mixed with a drop of *S.* Typhimurium cells on a microscope slide and rocked back and forth. To determine if the agglutination was due to type 1 fimbriae the same assay was performed in the presence of 0.5% D-mannose. If type 1 fimbriae were present on the *S.* Typhimurium cells they were agglutinated by the *S. cerevisae* in the absence but not the presence of mannose.

### Determination of Rate of Phase Variation

Rates of phase variation were calculated as previously described [25], [30], [39]. A single colony was resuspended in 1 ml of 0.85% NaCl. Serial dilutions were plated on EBU. After incubation at 37°C, the numbers of blue and light-colored colonies were counted. Using the formula (M/N)/g where ‘M/N’ is the ratio of light-colored colonies to the total number of colonies and ‘g’ is the number of generations of growth from a single cell to the total number of cells in the colony ((log10 number of cells/colony)/log_10_2), phase variation rates were calculated. The weighted average of the transition rates was calculated using the formula (M1/g1) + (M2/g2) + (Mn/gn))/(N1+N2+Nn) where M, N, and g are as above and n represents each individual phase variation rate calculation [25]. This gives a weighted rate of phase variation that takes into consideration the number of colonies used in each rate determination experiment.

An ampicillin enrichment procedure was used to identify phase variants when plating the i519, SL1344 and ATCC 14028 and is based on the discovery that only cells in the adhesive phenotype grew in LB containing no NaCl and thus were susceptible to killing by ampicillin. A single colony was grown in 3 ml of LB-0 medium containing ampicillin (100 ug/ml) for two hours at 37°C. This mixture was diluted and plated and the number of blue colonies found was used to determine a relative rate of phase variation. Mixtures also were plated before the addition of ampicillin to determine the number of cells in the colony. There generally was a ten-fold reduction in the number of viable cells after incubation with ampicillin.

### Tissue Culture and Invasion Assays

Henle 407 (ATCC CCL-6) cells (courtesy of J. Slauch) were maintained in Minimal Essential Medium with Earle’s Essential salts (MEM, Sigma), and 10% calf bovine serum. Cells were passaged every two to three days. Invasion assays were performed using a gentamicin protection assay [29]. Henle 407 cells were seeded in 24-well plates at ∼1×10^5^ cells per well. After growth overnight, cells were washed three times in phosphate buffered saline (PBS) to remove any trace antibiotics. Bacteria were grown overnight in high osmolarity LB (LB-H) without shaking. Overnight cultures were diluted 1∶10 into LB-H and 100 µl was inoculated into the cell culture wells. Plates were spun for 10 minutes at 100×g. Invasion proceeded for 30 min at 37°C with 5% CO_2_. Wells were rinsed with PBS and 1 ml of tissue culture media with gentamicin (100 µg/ml) was added. Plates were incubated for 1 hour at 37°C with 5% CO_2_. The cell monolayers were rinsed with PBS two times and lysed with 100–150 µl of 1% Triton X-100. Samples were mixed with 0.9 ml of LB-H. Bacteria were quantified by plating on appropriate media for CFU.

### Differential Gene Expression Using RNA-Seq

Strains SL1344 and the two phenotypic phase variants of strain 798 (i518 and i519) were grown in 5 ml LB broth overnight at 37°C. Overnight cultures were diluted 1∶100 in 10 ml LB and incubated at 37°C with shaking until mid logarithmic phase (OD_420_ = 0.7). Nine ml of culture was stabilized with 18 ml on RNA Protect (Qiagen, Valencia, CA). RNA was extracted after cell lysis and Proteinase K digestion using RNeasy Mini Kit (Qiagen, Valencia, CA). RNA was eluted from two columns with 30 µl of water and combined. RNA concentrations were measured with NanoDrop. 50 µl of each sample was then ethanol precipitated and disolved in 15 µl TE. mRNA was enriched using MicrobExpress Kit (Ambion, Grand Island, NY) following manufacturer’s recommendations. Final mRNA concentrations and purity was evaluated using Prokaryote Total RNA chip from Agilent. Samples were sequenced using an Illumina HiSeq 2000 sequencer (San Diego, CA). A separately prepared biological replicate for each sample was prepared and used in this analysis.

### Genome Sequencing

Total DNA was isolated from *S*. Typhimurium strain 798 using the DNEasy Kit (Qiagen, Valencia, CA). The genome was sequenced to a depth of 30× coverage using pyrosequencing on Roche 454 with Titanium technology (Branford, CT). Sequence reads were assembled into contigs with Newbler Assembler and was finished and checked for consistency using PCR [40]. The genome was annotated using the xBASE bacterial genome annotation service [41]. Gene predictions were performed using Glimmer [42], tRNA genes were identified with tRNAScan-SE [43], and ribosomal RNA genes were identified with RNAmmer [44]. Genomic comparisons were performed using MAUVE [45]. Single nucleotide polymorphisms were identified using SNPsFinder [46]. The completed sequence of strain 798 is deposited in Genbank under accession numbers CP003386 (chromosome) and CP003387 (plasmid).

### Data Analysis

Comparisons of rates of phase variation and invasion were analyzed for statistical significance in Excel (Microsoft, Inc., Redmond, WA) using the Student’s T test. The RNA-Seq data was analyzed using DEGseq (Bioconductor, Seattle, WA). Sequences were aligned using the published sequence for *S.* Typhimurium strain LT2 and the number of reads per gene was normalized as a percent of the total sequences obtained for that sample. The data was binned by gene and used as the measure of gene expression. Each strain had a biological duplicate. Criteria for significant differential gene expression was set at a p value of less than 0.05 and a minimum of a two-fold difference in expression. Comparisons were between i518 and i519 and i519 and SL1344.

## Supporting Information

Table S1 Comparison of expression of genes in S. Typhimurium i518 and i519 using RNA seq.
(XLSX)

Table S2 Comparison of expression of genes in S. Typhimurium i519 and SL1344 using RNA seq.
(XLSX)

Table S3 Comparison of expression of genes in S. Typhimurium i518 and SL1344 using RNA seq.
(XLSX)
